# Reduced Transplacental Transfer of Antimalarial Antibodies in Kenyan HIV-Exposed Uninfected Infants

**DOI:** 10.1093/ofid/ofz237

**Published:** 2019-05-20

**Authors:** Jessica E Ray, Katherine R Dobbs, Sidney O Ogolla, Ibrahim I Daud, John Vulule, Peter O Sumba, Rosemary Rochford, Arlene E Dent

**Affiliations:** 1 Center for Global Health and Diseases, Case Western Reserve University, Cleveland, Ohio; 2 Division of Pediatric Infectious Diseases, University Hospitals Rainbow Babies and Children’s Hospital, Cleveland, Ohio; 3 Centre for Global Health Research, Kenya Medical Research Institute, Kisumu, Kenya; 4 Department of Immunology and Microbiology, University of Colorado, Anschutz Medical Campus, Aurora, Colorado

**Keywords:** antimalarial antibodies, HIV-exposed uninfected neonate, inflammation, malaria, transplacental antibody transfer, vaccines

## Abstract

**Background:**

Altered neonatal immune responses may contribute to the increased morbidity observed in HIV-exposed but uninfected (HEU) infants compared with HIV-unexposed uninfected (HUU) infants. We sought to examine the effects of prenatal HIV and malaria exposure on maternal and neonatal plasma cytokine profiles and transplacental antibody transfer.

**Methods:**

Forty-nine HIV+ and 50 HIV- women and their HIV-uninfected neonate pairs from Kenya were assessed. All HIV+ mothers received combination antiretroviral therapy. Maternal plasma and cord blood plasma samples at delivery were tested for 12 cytokines, total IgG, and IgG specific to 4 vaccine antigens and 14 *Plasmodium falciparum* antigens.

**Results:**

HIV+ mothers had lower levels of all 12 plasma cytokines at delivery compared with HIV- mothers, but there were no differences between HEU and HUU neonates. There were no differences in the cord-to-maternal ratios (CMRs) of vaccine-specific IgG between HIV+/HEU and HIV-/HUU maternal–neonate pairs. HIV+/HEU maternal–neonate pairs had significantly lower CMRs for 3 antimalarial IgGs—merozoite surface protein 9, circumsporozoite protein, and erythrocyte binding antigen 181—which remained statistically significant after adjustment for malaria in pregnancy.

**Conclusions:**

In a cohort of optimally treated HIV-infected pregnant women, maternal HIV infection was associated with reduced transplacental transfer of antimalarial antibodies.

Prenatal HIV and malaria infection have each been associated with adverse pregnancy and birth outcomes, including maternal and fetal anemia, preterm birth, and low birth weight [1, 2]. Maternal HIV infection during pregnancy increases the risk of complications of malaria during pregnancy. *Plasmodium falciparum* (Pf) malaria during pregnancy, specifically placental malaria, may increase the risk of mother-to-child transmission of HIV, and the combination of both infections has the potential to cause significant perinatal morbidity and mortality [3].

With increased access to highly effective combination antiretroviral therapy (ART) and prevention of mother-to-child transmission (PMTCT) interventions implemented across sub-Saharan Africa, the number of infants infected with HIV is decreasing [4]. As the number of adults living with HIV increases and transmission to infants decreases, there is now a growing population of HIV-exposed but uninfected (HEU) infants [4]. HEU infants are healthier than HIV-infected infants [5], but various studies have suggested that HEU infants have greater morbidity and mortality than HIV-unexposed uninfected infants (HUU) [6]. HEU infants have been shown to have a greater frequency of all-cause sick clinic visits [7], greater infection rates [8], and a 1.2–2.7 times greater risk of hospitalization [9, 10] compared with HUU infants. Mortality rates for HEU infants have been shown to be 3–4 times greater than in HUU infants [5, 11]. The underlying mechanisms for these observations are likely multifactorial and may be influenced by parental illness and death, increased infectious exposures, impaired placental transfer of protective maternal antibodies, fetal antiretroviral therapy exposure, and subsequent immune perturbations.

Transplacental transfer of immunoglobulin G (IgG) from mother to fetus provides neonates with crucial immune protection early in life, especially in low-income countries where the burden of infectious diseases during infancy is high [12]. IgG is actively transferred across the placenta by binding the neonatal Fc receptor (FcRn) [12, 13]. Various factors have been associated with decreased transplacental transfer of maternal IgG, including structure of IgG subclasses [14, 15], glycosylation of IgG [16, 17], maternal total and specific antibody levels [18–20], maternal malnutrition [21], maternal noncommunicable diseases [22], and maternal infections such as HIV and malaria [23–27]. Neonatal IgG levels typically correlate with maternal levels. However, once maternal total IgG levels reach a threshold (>1500 mg/dL), the FcRn receptor becomes saturated, leading to less efficient transfer as IgG molecules compete for a finite number of receptors [12].

Most studies indicating increased HEU morbidity and mortality were conducted before lifelong ART was widely available to pregnant women in sub-Saharan Africa. In a cohort of HIV+ women receiving optimal ART, we aimed to understand the immunological consequences of prenatal HIV and malaria exposure on maternal–neonate pairs at the time of delivery through the investigation of birth anthropometric data, plasma cytokine profiles, transplacental transfer of antibodies to vaccine antigens, and naturally acquired infections such as malaria. Our results indicate that in optimally treated HIV+ women, defects in transplacental transfer of antimalarial antibodies persist.

## METHODS

### Ethical Approval

Informed consent was obtained from all participants in the appropriate local language. Ethical approval was obtained from the Institutional Review Board of University Hospitals Cleveland Medical Center (08-07-09), Cleveland, Ohio, the Colorado Multiple Institution Review Board (15–1277), and the Kenya Medical Research Institute Scientific and Ethical Review Unit (NON SSC 089).

### Study Site and Participants

The study was conducted at Chulaimbo County Hospital in Kisumu County, Kenya, between 2013 and 2015. Malaria transmission in this area is perennially high, with peaks coinciding with seasonal rains [28]. Chulaimbo Hospital serves a primarily rural catchment area outside of Kisumu and is an Academic Model Providing Access to Healthcare (AMPATH) site where clinical services and medications for HIV+ patients and their families are supported by USAID and the Indiana University–Kenya partnership. Participants included HIV- and HIV+ pregnant women who were enrolled at their first prenatal visit (typically during the second trimester) and followed throughout pregnancy (up to 4 prenatal visits). All HIV+ women received ART therapy. Fifty HIV- mothers/HUU neonates and 49 HIV+ mothers/HEU neonatal pairs with complete maternal and neonatal clinical data and samples at the time of delivery were included in this study. Data collected during prenatal visits included clinical history, physical exam, and concurrent diagnoses (eg, urinary tract infection, malaria). HIV- women received sulfadoxine-pyrimethamine (SP) beginning in the second trimester (for a total of 3 doses at intervals of 1 month or more). However, from January 2015 to November 2016, there was a shortage of SP at the clinic. HIV+ women did not receive SP, but they received cotrimoxazole as a part of their HIV treatment, which has antimalarial activity. Women were tested at their first prenatal visit for intestinal parasites including hookworm, *Trichuris trichiura, Ascaris lumbricoides, Entamoeba histolytica, Giardia lamblia, Strongyloides*, and *Schistosoma mansoni,* and, if positive, they were treated per Kenyan Ministry of Health guidelines. Neonatal data collected at delivery included APGAR scores, birth weight, length, and head circumference. Gestational age was calculated by the date of the last menstrual period.

### Blood Sample Collection and Processing

At delivery, cord blood and maternal venous blood samples were collected in heparinized syringes or vacutainers, and plasma was separated and stored at –80°C. All sample processing occurred within 1–5 hours of collection at the laboratory facilities at the Center for Global Health Research of KEMRI, located approximately 10 kilometers from Chulaimbo Hospital. All assays were conducted at the KEMRI laboratories.

### Detection of Pf infection by Polymerase Chain Reaction

From all time points available (up to 4 prenatal visits and delivery), DNA was extracted from up to 200 μL whole blood using Qiagen QIAmp DNA Mini Kits, as per the manufacturer’s instructions. Pf polymerase chain reaction (PCR) was performed as previously described [29]. Malaria in pregnancy was defined as infection detected by blood smear or by Pf PCR at any time. Previous studies of malaria in pregnancy have used blood smear, PCR, and/or placental histopathology at delivery to define malaria in pregnancy rather than detection of infection at any time during pregnancy. Of those women who were Pf PCR+, 52% had 1 time point that was positive, whereas 27% had 2 positive time points, 15% had 3 positive time points, 4% had 4 positive time points, and 2% had all 5 time points Pf PCR+.

### Measurement of Cytokines

We measured levels of 12 cytokines in plasma samples from 43 HIV-/HUU and 44 HIV+/HEU maternal–neonate pairs at the time of delivery. All plasma samples were assayed immediately after initial thawing. Some maternal/neonatal pairs were not included, as plasma had been previously thawed, which could compromise results. A multiplexed bead-based immunoassay was used to simultaneously measure plasma concentrations of interleukin (IL)-17F, interferon (IFN)-γ, IL-10, IL-12P70, IL-17A, IL-22, IL-1β, IL-21, IL-23, IL-6, IL-17E, and tumor necrosis factor alpha (TNFα) according to manufacturer instructions (Human Th17 Magnetic Bead Panel, EMD Millipore, Burlington, MA).

### Measurement of Total IGG

We measured total IgG in plasma samples from 41 HIV-/HUU and 40 HIV+/HEU maternal–neonate pairs at delivery using enzyme-linked immunosorbent assay (ELISA) (Human Total IgG ELISA Kits, AbCam, Cambridge, MA) following the manufacturer’s instructions, with these exceptions: (1) A 15-point standard curve was constructed using 1:2 serial dilutions, and (2) all cord blood and maternal samples were diluted 1:500 000 in assay buffer. Maternal and neonate pairs were run on the same plate to eliminate interplate variability. Plasma samples from some pairs were missing as they were consumed in other assays.

### Vaccine-Specific IgG

We measured IgG antibodies to diphtheria, tetanus, hepatitis B, and measles in plasma from maternal–neonate pairs at delivery by ELISA, as previously described [30, 31]. Multiple dilutions of samples were compared with 5-point standard curves made with serial dilutions from World Health Organization–approved antigen-specific reference sera; diphtheria Ig, human (NIBSC 10/262, 2 IU/mL), tetanus Ig, human (NIBSC TE-3, 120 IU/mL), hepatitis B Ig, human (NIBSC 07/164, 100 IU/mL), and measles Ig, human (NIBSC 97/648, 3 IU/mL).

### Pf Antigen-Specific IgG

We measured IgG antibodies to 14 recombinant Pf proteins in plasma samples from 48 HIV-/HUU and 46 HIV+/HEU maternal–neonate pairs at delivery using Luminex MagPix assays. Supplementary Table 1 contains Pf protein concentrations used in conjugation to magnetic microspheres. Pf proteins were conjugated to MagPix magnetic microspheres (MagPlex, Luminex, Austin, TX) according to the manufacturer’s instructions. For each assay, plasma was incubated with a master mix of Pf protein conjugated beads at a 1:1 ratio, for final plasma dilutions of 1:100 and 1:1000. R-Phycoerythrin-conjugated AffiniPure F(ab’) Fragment Goat Anti-Human IgG Fcγ Fragment Specific (Jackson ImmunoReaserch, West Grove, PA) was used as a secondary antibody. Seven malaria-naïve North American adult plasma samples were tested on all plates as negative controls. Mean fluorescent intensity (MFI) values were divided by the average MFIs of the negative controls. Final results are expressed as the fold-increase of the sample MFI relative to the negative control MFI (reported as fold over North American), as previously described [32].

### Statistical Analysis

For continuous numerical variables with normal distribution or n > 30, Student *t* tests were used. For continuous numerical variables with non-normal distribution or n < 30, Mann-Whitney tests or Kruskal-Wallis tests were performed. All basic statistical analyses were performed using Prism (GraphPad, La Jolla, CA) and JMP (SAS, Cary, NC) software. Multiple linear regression was performed to investigate the relationships between (1) maternal HIV status and maternal plasma cytokine levels, adjusting for effects of malaria and intestinal parasite infection in pregnancy, as well as age and gravidity; (2) maternal HIV status and maternal antimalarial IgG levels, adjusting for effect of malaria in pregnancy, age, and gravidity; and (3) maternal HIV status and CMR of antimalarial IgG, adjusting for effect of malaria in pregnancy, age, and gravidity. Variance inflation factor (VIF) values were calculated for each coefficient and were ≤6.1 in all models. Statistical significance was set at *P *< .05. All regression analyses were performed using base R [33].

## RESULTS

### Study Population

Fifty HIV- mothers/HUU neonates and 49 HIV+ mothers/HEU neonates were included in the study. The baseline characteristics and infections/infectious exposures of the cohort are summarized in Table 1. HIV+ women were older than HIV- women (mean age, 29 and 21 years, respectively; *P* < .0001) and more likely to be married (80% and 57%; *P* = .02). Additionally, HIV+ women were less likely to be primigravidae (*P* < .0001) and had higher gravidity than HIV- women (3.6 and 1.6; *P* < .0001). HIV+ women were less likely to have intestinal parasites during pregnancy (24% and 53%; *P* = .017) and less likely to have malaria during pregnancy (31% and 70%; *P* < .0001). There were no clinically significant differences in gestational age at delivery, birth weight, length, or head circumference between HUU and HEU neonates.

**Table 1.** Maternal Antenatal Characteristics and Infant Birth Characteristics

Maternal Antenatal Characteristics						
Mothers	HIV-	HIV+	*P* Value	No Malaria in Pregnancy	Malaria in Pregnancy	*P* Value
No.	50	49		49	50	
Age, y	21	29	<.0001	28	21.9	<.0001
Married, No. (%)	26/46 (57)	36/45 (80)	.02	32/42 (76)	30/49 (61)	.17
Primary school only, No. (%)	28/46 (61)	36/45 (80)	.07	31/42 (74)	33/49 (67)	.64
Gravidity	1.6	3.6	<.0001	3.3	1.9	<.0001
Primigravidae, No. (%)	23/43 (54)	3/43 (7)	<.0001	5/39 (11)	21/42 (50)	.0001
BMI, kg/m^2^	23.2	23.3	.8	23.7	22.8	.18
Hemoglobin, g/dL	11.3	10.5	.037	10.9	10.9	.9
Anemia (Hb < 11 g/dL), No. (%)	23/44 (52)	25/43 (58)	.67	23/44 (52)	25/43 (58)	.67
Intestinal parasite infection during pregnancy, No. (%)	21/40 (53)	8/33 (24)	.017	10/32 (31)	19/41 (46)	.23
Malaria in pregnancy, No. (%)	35/50 (70)	15/49 (31)	.0001			
HIV positive, No. (%)				34/49 (69)	15/50 (30)	.0001
Infant birth characteristics						
Infants	HUU	HEU	*P* Value	No Malaria in Pregnancy	Malaria in Pregnancy	*P* Value
No.	50	49		49	50	
Male, No. (%)	21/49 (43)	29/48 (60)	.1	25/47 (53)	25/50 (50)	.84
Gestational age, wk	38.6	37.7	.35	38.1	38.2	.98
Birth weight, kg	3.16	3.17	.86	3.24	3.1	.17
Low birth weight (<2.5 kg), No. (%)	6/49 (12)	8/48 (17)	.58	4/43 (9)	10/50 (20)	.15
Length, cm	46.9	47.4	.39	47.8	46.6	.02
Head circumference, cm	35.7	35	.08	35.3	35.5	.6

Abbreviations: BMI, body mass index; HEU, HIV-exposed but uninfected neonates; HUU, HIV-unexposed uninfected neonates.

### Lower Plasma Cytokines in HIV+ Compared With HIV- Mothers but no Differences Between HUU and HEU Neonates

**Figure 1.**
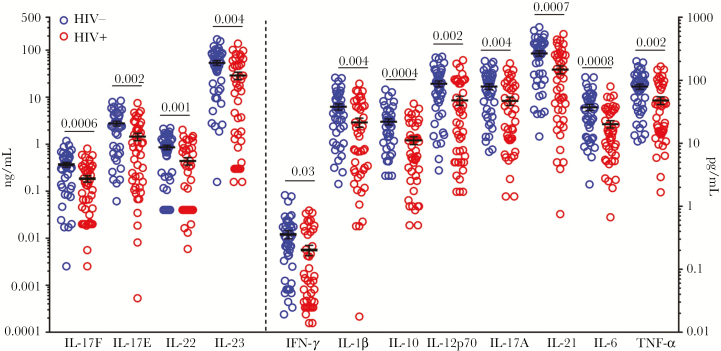
Lower plasma cytokine levels in HIV+ mothers compared with HIV- mothers. Maternal cytokines in plasma at delivery. Cytokines measured in 43 HIV- mothers (blue) and 44 HIV+ mothers (red). Cytokines to the left of the dotted line are associated with the left y-axis (ng/mL), and cytokines to the right of dotted line are associated with the right y-axis (pg/mL). All raw data points are plotted, with mean and SEM superimposed. The means of the 2 groups were compared using unpaired the Student *t* test. *P* values are presented for all statistically significant differences between the 2 groups.

**Figure 2.**
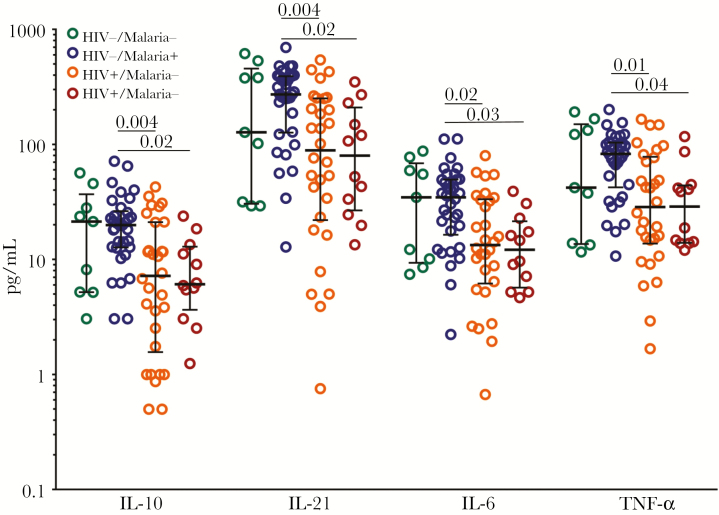
Lower plasma cytokines in HIV+ mothers with and without malaria in pregnancy. Maternal interleukin (IL)-10, IL-21, IL-6, and tumor necrosis factor in plasma at the time of delivery, measured in 9 HIV-/malaria- mothers (green), 34 HIV-/malaria+ mothers (blue), 30 HIV+/malaria- mothers (orange), and 12 HIV+/malaria+ mothers (maroon). All data points are plotted, and medians with interquartile ranges are superimposed. The Kruskal-Wallis test was used to compare medians across the 4 groups. *P* values are presented for all statistically significant differences among the groups.

To assess the degree of inflammation maternal infections could promote, we measured the concentrations of 12 cytokines (IL-17F, IFN-γ, IL-10, IL-12p70, IL-17A, IL-22, IL-1β, IL-21, IL-23, IL-6, IL-17E, and TNFα) in maternal and cord blood plasma at delivery in 43 HIV-/HUU and 44 HIV+/HEU maternal–neonate pairs. The mean concentrations of all 12 cytokines were significantly lower in HIV+ mothers than HIV- mothers (Figure 1). Mothers were then grouped based on HIV status and malaria infection during pregnancy (HIV-/malaria-, HIV-/malaria+, HIV+/malaria-, HIV+/malaria+). HIV+ mothers, both with and without malaria during pregnancy, still had lower median plasma cytokine values than HIV- women (Figure 2). The association between maternal HIV infection and lower maternal plasma cytokine levels remained statistically significant after adjustment for malaria in pregnancy, intestinal parasite infection in pregnancy, age, and gravidity (Supplementary Table 2). Intestinal parasite infection in pregnancy was independently associated with lower levels of IL-10. Malaria in pregnancy did not have a significant effect on maternal plasma cytokine levels. No difference was detected for any cytokine measured in cord blood between HUU and HEU neonates (Supplementary Figure 1).

### No Differences in Total IgG Between HIV- and HIV+ Mothers

We next measured total IgG levels in maternal and cord blood plasma at the time of delivery to assess hypergammaglobulinemia prevalence. No statistical difference in IgG levels between HIV- and HIV+ mothers was detected, nor was there a difference in IgG levels between HUU and HEU neonates (Supplementary Figure 2). The prevalence of hypergammaglobulinemia in mothers (defined as IgG >1600 mg/dL) was equally high in both HIV- (56.1%) and HIV+ (55%) women. Similarly, the prevalence of hypergammaglobulinemia in cord blood (defined as IgG >1400 mg/dL) was equally high in both HUU (29.3%) and HEU (20%) neonates.

To determine if there was an effect of malaria on total IgG levels, mothers were categorized based on both HIV status and malaria infection during pregnancy. No significant differences in maternal IgG levels were associated with maternal HIV or malaria infection (Supplementary Figure 3A). Neonates born to mothers of these categories also had no significant differences in cord blood IgG levels among the groups (Supplementary Figure 3B).

### No Differences in Vaccine-Specific IgG Levels Between HUU and HEU Neonates

**Figure 3.**
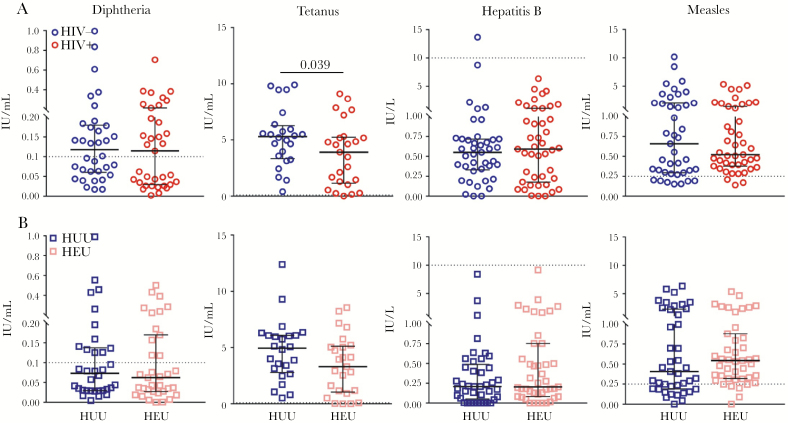
Vaccine-specific antibody levels in HIV- vs HIV+ mothers and HIV-unexposed uninfected (HUU) vs HIV-exposed but uninfected (HEU) neonates. A, Maternal antibody concentrations (circles) in HIV- (blue) and HIV+ (red) mothers. B, Corresponding neonatal antibody concentrations (squares) in HUU (navy blue) and HEU (pink) neonates. From left to right: diphtheria (n = 34 HIV-/HUU, n = 35 HIV+/HEU), tetanus (n = 24 HIV-/HUU, n = 25 HIV+/HEU), hepatitis B (n = 41 HIV-/HUU, n = 43 HIV+/HEU), and measles (n = 41 HIV-/HUU, n = 43 HIV+/HEU). Horizontal dotted lines represent antibody concentrations considered to be protective against infection (diphtheria, 0.1 IU/mL; tetanus, 0.1 IU/mL; hepatitis B, 10 IU/L; and measles, 0.25 IU/mL) All data points are plotted, and medians with interquartile ranges are superimposed. Medians were compared for each antibody using the Mann-Whitney test. *P* values are presented for statistically significant differences.

Vaccine-specific IgG antibodies to 4 vaccines—diphtheria, tetanus, hepatitis B, and measles—were measured in HIV-/HUU and HIV+/HEU maternal–neonate pairs at the time of delivery. There were no significant differences in the median antibody levels of HIV- and HIV+ mothers for diphtheria, hepatitis B, or measles. HIV- women had significantly higher tetanus antibody levels than HIV+ women (*P* = .039) (Figure 3A). There were no differences in vaccine antibody levels between HUU and HEU neonates (Figure 3B). To measure transplacental transfer of maternal IgG to her neonate, CMRs were calculated for each maternal–neonate pair for each vaccine-specific antibody. There were no statistically significant differences in the CMRs of HIV-/HUU and HIV+/HEU maternal–neonate pairs (Supplementary Figure 4), suggesting no effect of maternal HIV infection on transplacental transfer of vaccine-specific IgG.

### Decreased Transplacental Transfer of Antimalarial Antibodies in HIV+/HEU Maternal–Neonate Pairs

**Figure 4.**
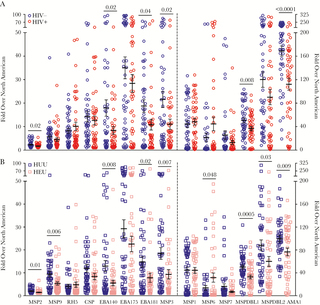
Lower antimalarial antibody levels in HIV+ vs HIV- mothers and in HIV-exposed but uninfected (HEU) vs HIV-unexposed uninfected (HUU) neonates. Antibodies to 14 Pf-specific antigens measured in maternal plasma at the time of delivery and in cord blood plasma. A, 48 HIV- mothers (blue) and 46 HIV+ mothers (red) and (B) 48 HUU neonates (navy blue) and 46 HEU neonates (pink) included. All data points are plotted, and means with SEMs are superimposed. All antigen-specific antibodies to the left of the dotted line are plotted against the left y-axis; all antigen-specific antibodies to the right of the dotted line are plotted against the right y-axis. An unpaired Student *t* test was used to compare the means of values for HIV- vs HIV+ mothers and for HUU vs HEU neonates. *P* values for all statistically significant differences are shown.

Fourteen Pf antigen-specific IgG antibodies (MSP2, MSP9, PfRh5, CSP, EBA141, EBA175, EBA180, MSP3, MSP1, MSP6, MSP7, MSPDBL1, MSPDBL2, and AMA1) were measured in 48 HIV-/HUU and 46 HIV+/HEU maternal–neonate pairs. Pre-erythrocytic (CSP) and erythrocytic antigen targets were chosen based on prior studies associating antibody responses with protection from malaria [32, 34]. HIV+ mothers generally had lower antibody levels than HIV- mothers, and these were statistically significantly lower for MSP2, EBA140, EBA181, MSP3, MSPDBL1, and AMA1 (Figure 4A). After adjustment for malaria in pregnancy, age, and gravidity, the relationship between maternal HIV infection and antimalarial antibody levels was no longer statistically significant, except for EBA181 and AMA1 (Supplementary Table 3). For MSP2, EBA140, MSP3, and MSPDBL1, higher antibody levels were associated with malaria in pregnancy, whereas HIV infection showed no statistically significant relationship in multivariable analysis. Thus, the lower antimalarial antibody levels in HIV+ mothers observed in univariate analysis were likely related to lower rates of malaria in pregnancy and differences in age and gravidity for HIV+ mothers.

**Figure 5.**
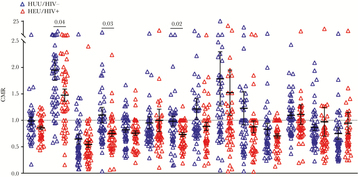
Lower cord-to-maternal ratios of MSP9, CSP, and EBA181 in HIV+/ HIV-exposed but uninfected (HEU) maternal–neonate pairs. Cord-to-maternal ratios (CMRs) of antibodies to 14 Pf antigens. Forty-eight HIV-/ HIV-unexposed uninfected (HUU) CMRs (blue) and 46 HIV+/HEU CMRs (red) are presented. Each data point represents a maternal–neonate pair; means with SEMs are superimposed. An unpaired Student *t* test was used to compare ratio means of HIV-/HUU and HIV+/HEU. *P* values for all statistically significant differences are shown.

We next evaluated Pf antibody levels in cord blood. HEU neonates generally had lower antimalarial antibody levels than HUU neonates with statistically significantly lower antibody levels for MSP2, MSP9, EBA140, EBA181, MSP3, MSP6, MSPDBL1, MSPDBL2, and AMA1 (Figure 4B). Finally, to assess transplacental transfer of maternal antimalarial IgG to neonates, CMRs were calculated for each maternal–neonate pair. Generally, CMRs of HIV+/HEU maternal–neonate pairs were lower than CMRs of HIV-/HUU pairs, suggesting decreased transplacental antimalarial antibody transfer in HIV+/HEU pairs compared with HIV-/HUU pairs, with statistically significantly lower CMRs for MSP9 (*P* = .04), CSP (*P* = .03), and EBA181 (*P* = .017) (Figure 5). The association between maternal HIV infection and decreased transplacental transfer of antimalarial antibodies remained statistically significant after adjustment for malaria in pregnancy, age, and gravidity (Supplementary Table 4). For CSP IgG, both HIV and malaria in pregnancy were associated with significantly decreased transplacental antibody transfer. There were no other statistically significant differences in transplacental transfer of antimalarial antibodies between mothers with and without malaria in pregnancy. Although CMRs of vaccine antibodies were not affected by HIV or malaria status, lower CMRs of antibodies important to naturally acquired malaria infection were observed in HIV+/HEU pairs, revealing an important effect even in optimally treated HIV.

## DISCUSSION

In this maternal–neonate cohort study of HIV+ women on ART and HIV- women living in a malaria-endemic area of western Kenya, we found no effect of maternal HIV or malaria infection in pregnancy on birth outcomes. Although HIV+ mothers had lower inflammatory plasma cytokines at delivery, there were no differences in cord blood plasma cytokines according to prenatal HIV or malaria exposure. We found no effect of maternal HIV or malaria infection on transplacental transfer of vaccine-specific antibodies. In contrast, maternal HIV infection was associated with reduced transplacental transfer of a subset of antimalarial antibodies that remained significant after adjusting for maternal malaria infection. Similar levels of total IgG were seen in HIV- and HIV+ mothers, suggesting that the effect of HIV on transplacental transfer of antimalarial antibodies involves mechanisms independent of hypergammaglobulinemia.

HIV+ mothers had lower inflammatory cytokines than HIV- mothers in this study, which may be attributed to the HIV+ mothers taking cotrimoxazole during pregnancy. Cotrimoxazole not only prevents malaria but has been found to reduce systemic inflammation by either indirectly targeting pathogens or modulating cells that produce pro-inflammatory cytokines. HIV+ adults taking cotrimoxazole have been shown to have reduced plasma inflammatory biomarkers [35, 36] and 1 study indicated that cotrimoxazole reduces systemic inflammation in HIV infection by altering the gut microbiome and immune activation [37].

Studies conducted before optimal ART therapy in pregnancy found that HEU infants had lower birth weights compared with HUU infants [5, 38]. Longitudinal data from a large HIV cohort in the United States showed that increased use of maternal ART coincided with a decline in low–birth weight HIV-exposed neonates [39]. In our cohort and those studied after optimal maternal ART, the majority of HEU neonates had normal birth weight [40]. A recent study of Brazilian HIV+ mothers on optimal ART therapy found no difference in birth outcomes or in cord blood cytokine levels in HEU vs HUU neonates [41]. Thus, the lack of association with HIV exposure and adverse birth outcomes in our cohort likely reflects optimally controlled maternal HIV infection.

Previous studies showed that HEU infants have higher morbidity and mortality early in life, specifically susceptibility to infectious diseases, compared with HUU infants [6]. Although likely multifactorial, decreased transplacental transfer of protective maternal antibodies probably contributes. Multiple studies have demonstrated reduced levels of pathogen-specific antibodies in HIV+ mothers, with reduced CMRs specific to *Streptococcus pneumonia*, *Haemophilus influenza*e type b, group B *Streptococcus *(GBS), tetanus, measles, and polio (reviewed in [23]). Most of these studies were conducted before widespread ART. Bosire et al. found that transplacental transfer of antibodies against measles, pneumococcus, and rotavirus was higher in HIV+ women on triple ART compared with HIV+ women on short-course zidovudine alone, underscoring the positive impact of optimal ART [42]. Recent studies have examined transplacental antibody transfer in HIV+ women treated with more optimal ART in pregnancy and found persistent reduced CMR to respiratory syncytial virus (RSV) and GBS compared with HIV- women [43–45].

With regards to transplacental transfer of antimalarial antibodies, Ayisi et al. found that Kenyan HIV+ women (receiving no ART) had reduced transfer of antibodies against only CSP (NANP)_5_ but not antibodies against MSP1, EBA175, or against peptides of MSP2/MSP3 or tetanus [46]. In contrast, Moro et al. found that HIV+ Mozambican women (receiving no ART) had reduced CMR of antibodies against MSP1, Pf lysate, AMA1, and EBA175 [26]. HIV+ Cameroonian women who received only nevirapine at delivery had decreased CMRs to CSP, AMA1, MSP1, and tetanus compared with HIV- women [47]. This was thought to be driven by elevated hypergammaglobulinemia in HIV+ women, but after adjusting for this, decreased CMR of CSP, MSP1, and tetanus persisted. To our knowledge, our study is the first to specifically examine transplacental transfer of antimalarial antibodies in optimally treated HIV+ women in resource-limited settings.

Malaria in pregnancy is associated with reduced transplacental transfer of antibodies to tetanus, Epstein-Barr virus, herpes simplex virus, varicella zoster virus, and RSV [18, 27, 48] and with reduced transfer of antibodies to EBA175, AMA1, and MSP1 [26]. HIV and malaria in pregnancy both drive hypergammablobulinemia, which independently has been associated with reduced transfer of antibodies to pathogens such as RSV [43, 49].

Mechanisms underlying reduced transplacental transfer in perinatal infections are poorly understood but may include placental inflammation and induction of hypergammaglobulinemia, leading to saturation of the placental FcRn [12]. Other factors that may contribute include variations in the structure of IgG subclasses (IgG1 is preferentially transported, followed by IgG4, IgG3, and IgG2) [14], IgG subclass polymorphisms that affect FcRn binding [13], and IgG glycosylation patterns [12]. Further studies are needed to better understand the complex factors leading to the reduced transplacental transfer associated with perinatal infections.

A limitation of this study is that we did not measure IgG subclasses to vaccine or Pf antigens, which might have increased our ability to detect differences in CMRs according to maternal HIV or malaria infection, as observed in some studies that detected significant differences in antimalarial IgG1 and IgG3 CMRs but not in total antimalarial IgG [26, 47]. Additionally, baseline differences between HIV+ and HIV- women (most notably age and gravidity) affect their risk for malaria during pregnancy in that younger and primi- or secundi-gravidae women are at highest risk [50].

In conclusion, differences in clinical and immune characteristics between HIV+ and HIV- mothers were subtle, and in some respects and perhaps unexpectedly, HIV+ mothers had healthier pregnancies, as evidenced by lower proportions with intestinal parasites or malaria. Importantly, this is one of the first studies to examine birth outcomes and immune characteristics among HEU infants in which the standard of care for HIV+ pregnant women included highly effective lifelong ART. These results highlight the importance of providing high-quality care to all HIV-infected women throughout pregnancy and postpartum to prevent transmission to infants and to optimize clinical outcomes for mother and child. Vaccines with powerful adjuvants may overcome subtle immunodeficiencies that are better revealed and investigated through natural infection immune responses, potentially revealing novel mechanisms of HIV pathogenesis.

## Supplementary Data

Supplementary materials are available at *Open Forum Infectious Diseases* online. Consisting of data provided by the authors to benefit the reader, the posted materials are not copyedited and are the sole responsibility of the authors, so questions or comments should be addressed to the corresponding author.

ofz237_suppl_supplementary_figures

ofz237_suppl_supplementary_tables
