# Supplementary material for: Reduced Transplacental Transfer of Antimalarial Antibodies in Kenyan HIV-Exposed Uninfected Infants
Source: Open Forum Infect Dis. 2019 May 20;6(6):ofz237. doi: 10.1093/ofid/ofz237 (PMC6563943; doi:10.1093/ofid/ofz237)
Supplement: ofz237_suppl_supplementary_figures [file ofz237_suppl_supplementary_figures.pdf]

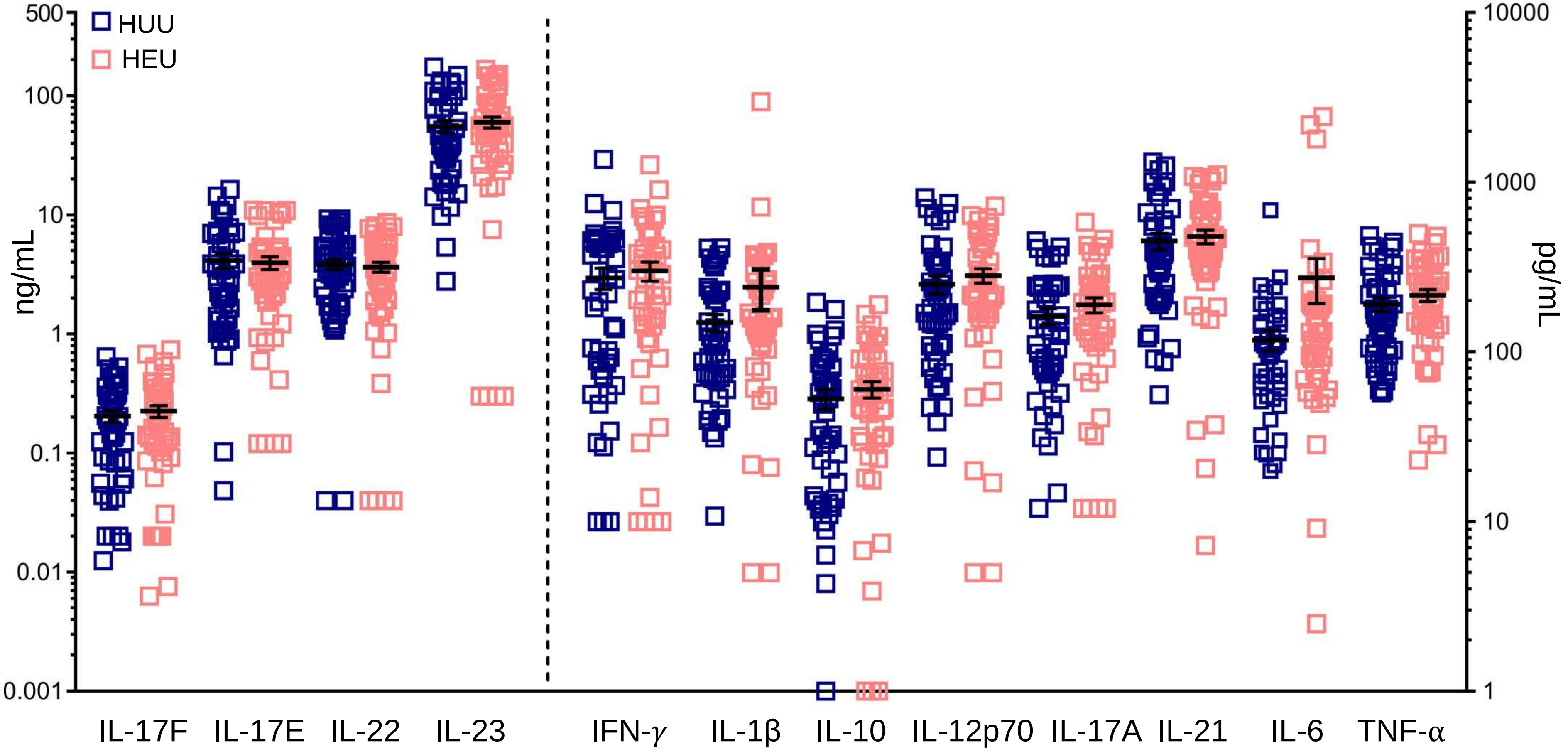

Supplemental Figure 1. No differences in plasma cytokine levels between HUU and HEU neonates. Neonatal cytokines in cord blood plasma measured in 43 HUU (blue) and 44 HEU (pink) neonates. Cytokines left of the dotted line are associated with the left y axis (ng/mL) and cytokines right of dotted line are associated with the right y axis (pg/mL). Individual values plotted and mean with SEM superimposed. Unpaired t-test used to compare means of HUU and HEU neonates for each cytokine. No significant differences detected.

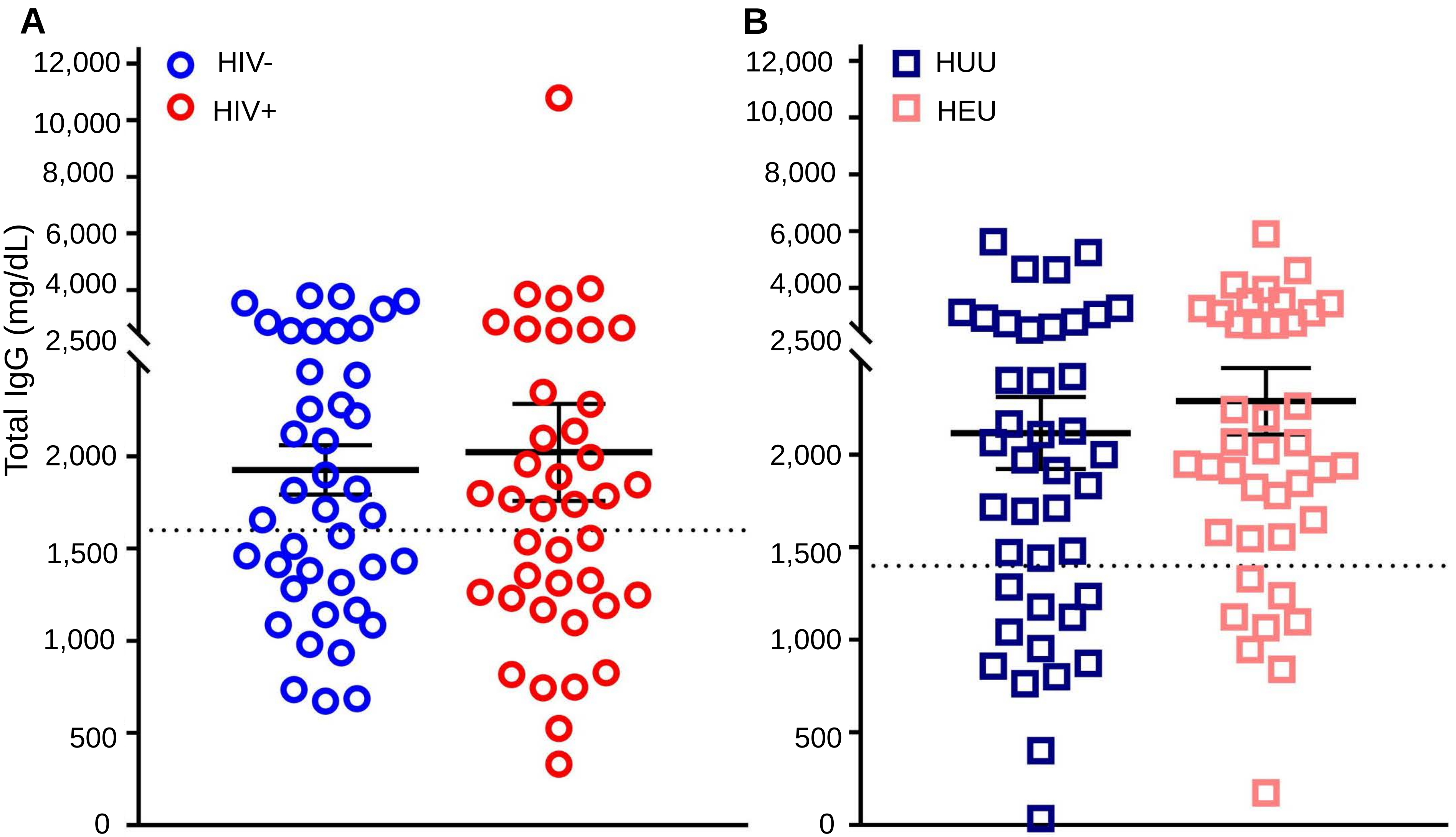

Supplemental Figure 2. No differences in total IgG levels between HIV- and HIV+ mothers and between HUU and HEU neonates. Total IgG (mg/dL) in maternal plasma and neonatal cord blood plasma at time of delivery. (A) Maternal total IgG in 41 HIV- women (blue) and 40 HIV+ women (red). All data points plotted and means with SEM superimposed**.** Horizontal dotted line represents threshold of hypergammaglobulinemia (>1600 mg/dL). (B) Neonatal total IgG in 41 HUU infants (navy blue) and 40 HEU infants (pink). All data points plotted and means with SEM superimposed. Horizontal line represents threshold of hypergammaglobulinemia for infants 0-1 year of age (>1400 mg/dL). Means of the two groups compared using unpaired student t-tests with no significant difference detected.

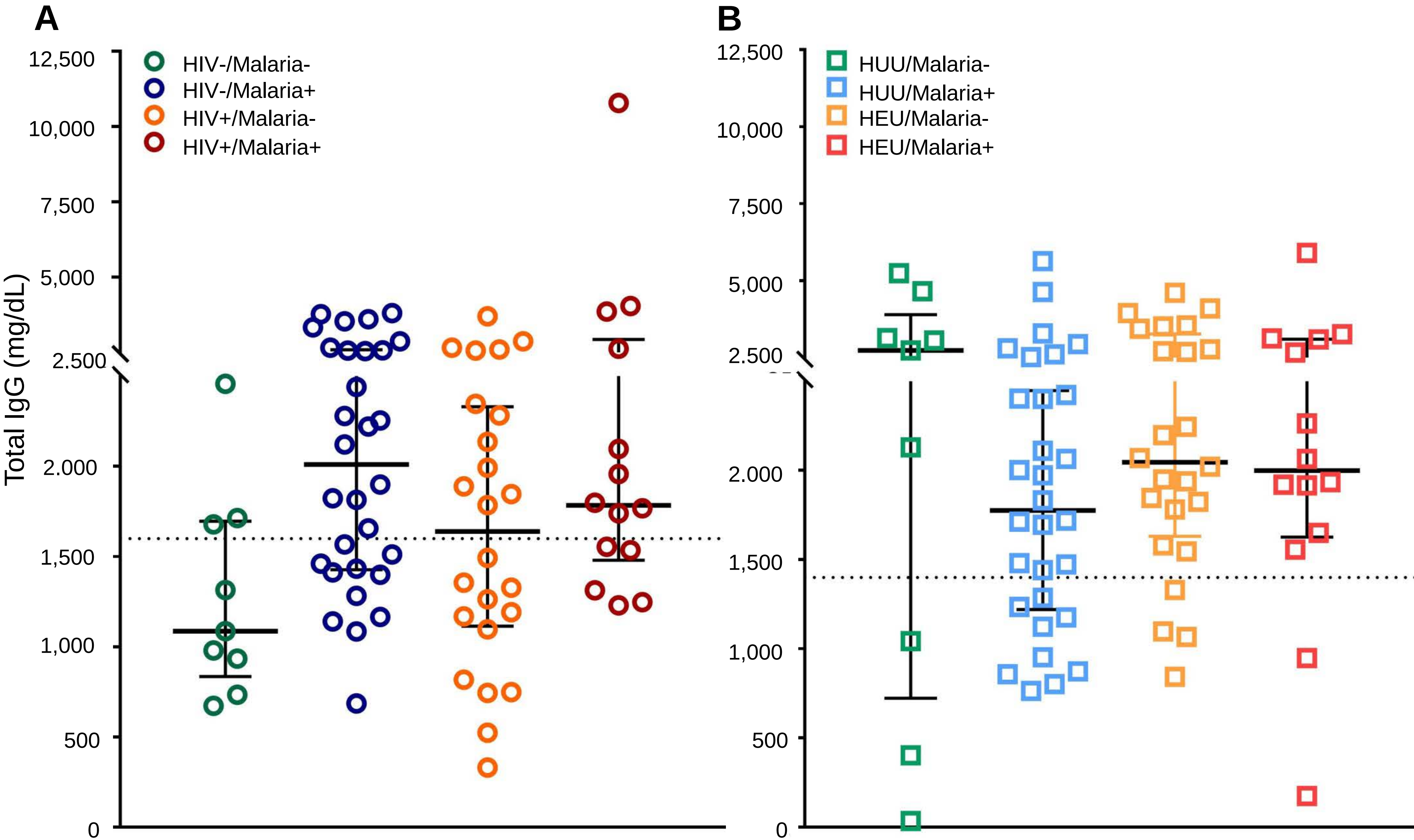

Supplemental Figure 3. No differences in maternal or neonatal total IgG levels according to maternal HIV status or malaria in pregnancy. Total IgG (mg/dL) present in maternal and neonatal cord blood plasma at time of delivery in four maternal infection exposure groups. (A) Maternal total IgG (circles), 9 HIV-/malaria- (green), 30 HIV-/malaria+ (dark blue), 24 HIV+/malaria- (orange), and 14 HIV+/malaria + (maroon). All data points plotted and medians with interquartile range superimposed. Horizontal dotted line represents threshold of hypergammaglobulinemia (>1600 mg/dL). Kruskal-Wallis test used to compare medians across the four groups with no significant differences detected. (B) Neonatal total IgG (squares), 9 HUU/malaria- (light green), 30 HUU/malaria+ (light blue), 24 HEU/malaria- (orange), and 14 HEU/malaria + (red). All data points plotted with medians with interquartile range superimposed. Horizontal line represents threshold of hypergammaglobulinemia for infants 0-1 year of age (>1400 mg/dL). Kruskal-Wallis test used to compare medians across the four groups with no significant differences detected.

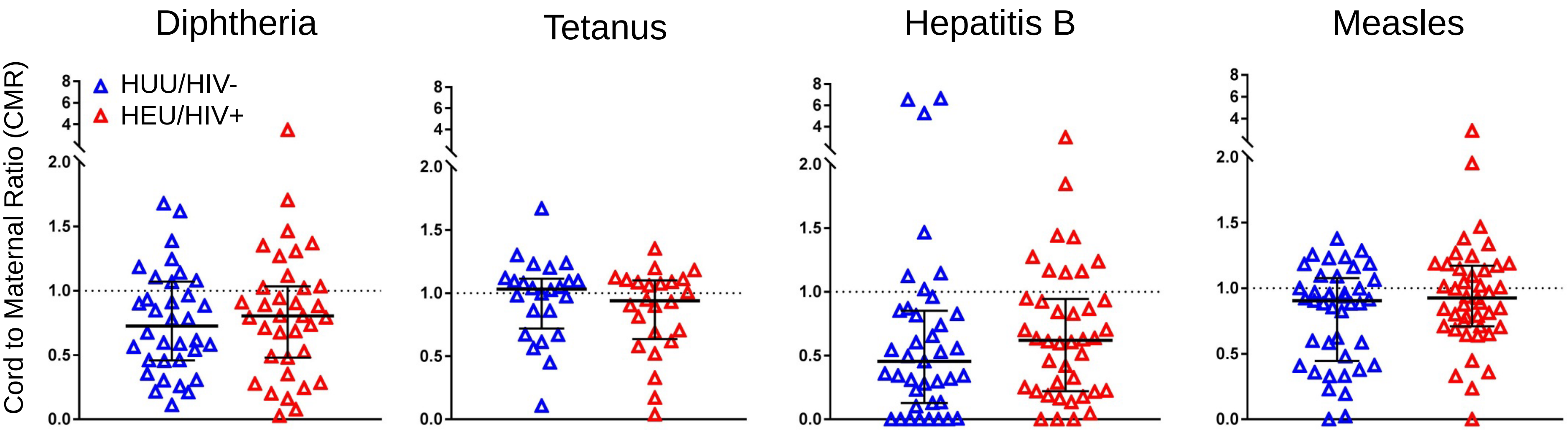

Supplemental Figure 4. No differences in cord-to-maternal ratios (CMRs) of diphtheria, tetanus, hepatitis B, and measles between HIV-/HUU and HIV+/HEU maternal-neonate pairs. CMRs of four vaccine antibodies for HIV-/HUU (blue) and HIV+/HEU (red) pairs for diphtheria CMR (n = 34 HIV-/HUU, n = 35 HIV+/HEU), tetanus CMR (n = 24 HIV-/HUU, n = 25 HIV+/HEU), hepatitis B CMR (n = 41 HIV-/HUU, n = 43 HIV+/HEU), and measles CMR (n = 41 HIV-/HUU, n = 43 HIV+/HEU). All data points plotted and medians with interquartile range superimposed. Medians of the two groups compared for each antibody using Mann-Whitney test with no significant differences detected.
