# Supplementary material for: Reduced Transplacental Transfer of Antimalarial Antibodies in Kenyan HIV-Exposed Uninfected Infants
Source: Open Forum Infect Dis. 2019 May 20;6(6):ofz237. doi: 10.1093/ofid/ofz237 (PMC6563943; doi:10.1093/ofid/ofz237)
Supplement: ofz237_suppl_supplementary_tables [file ofz237_suppl_supplementary_tables.docx]

**Supplementary Table 2. Multiple regression estimates of the effects of maternal HIV, malaria in pregnancy, intestinal parasite infection in pregnancy, age, and gravidity on maternal plasma cytokine levels.** IFN-𝛾 was natural log transformed. n = 64 for all models.

| **Maternal**  **cytokine** | **Predictor** | **ß** | **95% CI** | ***p* value** |
| --- | --- | --- | --- | --- |
| IL-17F | HIV | **-0.2** | **-0.3, -0.01** | **0.03** |
|  | Malaria in pregnancy | -0.03 | -0.2, 0.1 | 0.7 |
|  | Intestinal parasite infection  Age  Gravidity | -0.1  0.09  -0.2 | -0.2, 0.03  -0.2, 0.4  -0.5, 0.1 | 0.1  0.5  0.2 |
|  |  |  |  |  |
| IL-17E | HIV | **-1.4** | **-2.8, -0.1** | **0.04** |
|  | Malaria in pregnancy | -0.3 | -1.5, 0.8 | 0.6 |
|  | Intestinal parasite infection  Age  Gravidity | -0.6  1.7  -2.4 | -1.7, 0.5  -0.7, 4.1  -4.9, 0.03 | 0.3  0.2  0.05 |
|  |  |  |  |  |
| IL-22 | HIV | **-0.4** | **-0.8, -0.004** | **0.048** |
|  | Malaria in pregnancy | -0.08 | -0.4, 0.2 | 0.6 |
|  | Intestinal parasite infection  Age  Gravidity | -0.2  0.4  -0.7 | -0.5, 0.1  -0.3, 1.1  -1.4, 0.01 | 0.2  0.2  0.05 |
|  |  |  |  |  |
| IL-23 | HIV | **-27** | **-53, -1.5** | **0.04** |
|  | Malaria in pregnancy | -6.5 | -29, 16 | 0.6 |
|  | Intestinal parasite infection  Age  Gravidity | -15.5  24  -40 | -37, 5.8  -22, 69  -88, 6.9 | 0.15  0.3  0.09 |
|  |  |  |  |  |
| IFN-𝛾 | HIV | **-0.9** | **-1.7, -0.1** | **0.03** |
|  | Malaria in pregnancy | -0.1 | -0.8, 0.6 | 0.8 |
|  | Intestinal parasite infection  Age  Gravidity | -0.5  0.3  -0.8 | -1.2, 0.2  -1.2, 1.7  -2.3, 0.8 | 0.2  0.7  0.3 |
|  |  |  |  |  |
| IL-1ß | HIV | **-18** | **-35, -0.3** | **0.046** |
|  | Malaria in pregnancy | -5.6 | -21, 9.6 | 0.5 |
|  | Intestinal parasite infection  Age  Gravidity | -11  11  -23 | -26, 3  -19, 42  -55, 8.4 | 0.1  0.5  0.1 |
|  |  |  |  |  |
| IL-10 | HIV | **-13.2** | **-22, -4.3** | **0.004** |
|  | Malaria in pregnancy | 0.3 | -7.5, 8.1 | 0.93 |
|  | Intestinal parasite infection  Age  Gravidity | **-7.6**  -3.2  -3.2 | **-15, -0.3**  -19, 12  -19, 13 | **0.042**  0.7  0.7 |
|  |  |  |  |  |
| IL-12p70 | HIV | **-46** | **-84, -8.7** | **0.02** |
|  | Malaria in pregnancy | -10 | -43, 22 | 0.5 |
|  | Intestinal parasite infection  Age  Gravidity | -22  29  -55 | -53, 8.5  -37, 95  -123, 14 | 0.2  0.4  0.1 |
|  |  |  |  |  |
| IL-17A | HIV | **-36** | **-70, -2.7** | **0.03** |
|  | Malaria in pregnancy | -12 | -41, 17 | 0.4 |
|  | Intestinal parasite infection  Age  Gravidity | -17  34  -56 | -45, 10  -25, 93  -118, 5.2 | 0.2  0.2  0.07 |
|  |  |  |  |  |
| IL-21 | HIV | **-127** | **-231, -22** | **0.02** |
|  | Malaria in pregnancy | -25 | -116, 66 | 0.6 |
|  | Intestinal parasite infection  Age  Gravidity | -59  103  -172 | -145, 27  -81, 288  -364, 19 | 0.2  0.3  0.1 |
|  |  |  |  |  |
| IL-6 | HIV | **-19** | **-35, -3.2** | **0.02** |
|  | Malaria in pregnancy | -6.6 | -20, 7.3 | 0.3 |
|  | Intestinal parasite infection  Age  Gravidity | -10  1.6  -15 | -23, 2.8  -26, 30  -44, 14 | 0.12  0.9  0.3 |
|  |  |  |  |  |
| TNF | HIV | **-36** | **-67, -6.3** | **0.02** |
|  | Malaria in pregnancy | -11 | -37, 15 | 0.4 |
|  | Intestinal parasite infection  Age  Gravidity | -19  31  -51 | -44, 6  -22, 84  -106, 3.8 | 0.1  0.2  0.1 |
|  |  |  |  |  |
|  |  |  |  |  |

**Supplementary Table 3. Multiple regression estimates of the effects of maternal HIV, malaria in pregnancy, age, and gravidity on maternal antimalarial IgG levels.** MSP2, EBA140, EBA181, and MSP3 were natural log transformed. n = 94 for all models.

| **Maternal anti-malarial IgG** | **Predictor** | **ß** | **95% CI** | ***p* value** |
| --- | --- | --- | --- | --- |
| MSP2 | HIV | -0.2 | -0.6, 0.2 | 0.3 |
|  | Malaria in pregnancy  Age  Gravidity | **0.5**  -0.5  0.6 | **0.2, 0.9**  -1.1, 0.2  -0.1, 1.3 | **0.003**  0.1  0.1 |
|  |  |  |  |  |
| EBA140 | HIV | -0.2 | -0.9, 0.5 | 0.6 |
|  | Malaria in pregnancy  Age  Gravidity | **0.97**  -1  0.8 | **0.3, 1.6**  -2.2, 0.2  -0.4, 2.1 | **0.003**  0.1  0.2 |
|  |  |  |  |  |
| EBA181 | HIV | **-0.8** | **-1.6, -0.004** | **0.049** |
|  | Malaria in pregnancy  Age  Gravidity | **0.9**  -1.1  **1.5** | **0.2, 1.6**  -2.4, 0.2  **0.1, 2.9** | **0.008**  0.1  **0.03** |
|  |  |  |  |  |
| MSP3 | HIV | -0.5 | -1.2, 0.2 | 0.2 |
|  | Malaria in pregnancy  Age  Gravidity | **0.6**  **-1.6**  **1.4** | **-0.004, 1.2**  **-2.8, -0.5**  **0.1, 2.6** | **0.05**  **0.006**  **0.03** |
|  |  |  |  |  |
| MSP DBL1 | HIV | -9.9 | -23, 3.5 | 0.1 |
|  | Malaria in pregnancy  Age  Gravidity | **12.2**  **-22**  21 | **0.3, 24**  **-45, 0.3**  -2.5, 45 | **0.045**  **0.05**  0.08 |
|  |  |  |  |  |
| AMA1 | HIV | **-40** | **-73, -6.4** | **0.02** |
|  | Malaria in pregnancy | **37** | **7.5, 66** | **0.01** |
|  | Age  Gravidity | -34  28 | -90, 21  -31, 87 | 0.2  0.4 |
|  |  |  |  |  |

**Supplementary Table 4. Multiple regression estimates of the effects of maternal HIV, malaria in pregnancy, age, and gravidity on trans-placental transfer of antimalarial IgG.** MSP9 was natural log transformed. n = 94 for all models.

| **Antigen, Cord:Maternal Ratio (CMR)** | **Predictor** | **ß** | **95% CI** | ***p* value** |
| --- | --- | --- | --- | --- |
| MSP9 | HIV | **-0.3** | **-0.6, -0.04** | **0.02** |
|  | Malaria in pregnancy  Age  Gravidity | 0.1  -0.2  0.4 | -0.1, 0.4  -0.7, 0.2  -0.1, 0.9 | 0.3  0.3  0.2 |
|  |  |  |  |  |
| CSP | HIV | **-0.4** | **-0.8, -0.03** | **0.04** |
|  | Malaria in pregnancy  Age  Gravidity | **-0.4**  0.04  -0.2 | **-0.8, -0.1**  -0.6, 0.7  -0.9, 0.5 | **0.02**  0.9  0.6 |
|  |  |  |  |  |
| EBA181 | HIV | **-0.3** | **-0.6, -0.06** | **0.02** |
|  | Malaria in pregnancy  Age  Gravidity | -0.2  -0.02  -0.004 | -0.4, 0.03  -0.4, 0.4  -0.5, 0.5 | 0.1  0.9  0.99 |
